# Editorial: Rhizophagy and other cross-talks in rhizobiocomplex

**DOI:** 10.3389/fmicb.2025.1763865

**Published:** 2026-01-19

**Authors:** Nagendran Rajalingam, Abhinav Aeron, Verinder Virk, Tofazzal Islam

**Affiliations:** 1 Center for Food Biotechnology and Microbiology, Ghent University Global Campus, Incheon, Republic of Korea; 2 Division of Biotechnology, College of Environmental and Bioresource Sciences, Jeonbuk National University, Iksan, Republic of Korea; 3 Faculty of Sciences, Gurukul Kangri University, Haridwar, India; 4 Institute of Biotechnology and Genetic Engineering (IBGE), Gazipur Agricultural University, Gazipur, Bangladesh

**Keywords:** nutrient acquisition, plant-microbe cross-talk, rhizobiocomplex, rhizobiome engineering, rhizophagy, root-microbe interaction

The rhizosphere is a complex, highly dynamic biological interface in the soil immediately surrounding plant roots. This zone, often termed the rhizobiocomplex, functions similarly to the gut microbiota in animals, characterized by co-evolved interactions between the roots, soil, and a diverse microbial community. Within this critical zone, approximately 100 μm around the rhizoplane (root surface), dynamic cross-talk mechanisms are mediated by the exchange of root exudates and microbial metabolites (Islam et al., 2005). These biochemical signals govern microbial recruitment, metabolism, and the overall functional integrity of the system.

## The rhizophagy cycle: a novel mechanism for nutrient acquisition

A compelling and relatively recent concept in this field is rhizophagy (literally, “root eating”). This term describes a cyclic biological process in which host plant root cells temporarily internalize certain non-pathogenic microorganisms (e.g., bacteria and fungi) to extract nutrients (Paungfoo-Lonhienne et al., 2013). Initial proof-of-concept experiments demonstrated that model plants like *Arabidopsis thaliana* and *Lycopersicum esculentum* (tomato) could internalize non-pathogenic microbes, including *Escherichia coli* and *Saccharomyces cerevisiae*, into their root cells, effectively digesting them as a nutrient source (Paungfoo-Lonhienne et al., 2010).

The current model describes a rhizophagy cycle where microbes acquire soil nutrients during their free-living soil phase. Upon internalization into the root cells (the intracellular/endophytic phase), nutrients are extracted via a process involving oxidative stress (White et al., 2018). This oxidative mechanism forces microbes to release essential nutrients (e.g., nitrogen, phosphorus, zinc) into the plant tissue. Surviving microbes, often lacking cell walls, are expelled through growing root hairs to re-enter the soil and continue gathering nutrients. While rhizophagy represents a paradigm shift in understanding plant nutrition (Mata et al., 2025), the literature remains limited, partly due to the experimental complexity of studying these dynamic, intracellular events (Zhang et al., 2022).

## Integrating rhizophagy into the rhizobiocomplex framework

The articles in this Research Topic, though not exclusively centered on rhizophagy or the rhizobiocomplex, collectively advance our understanding of how plant performance (including nutrition, stress tolerance, and metabolic specialization) relies on the coordinated, multi-faceted activities within the entire rhizobiocomplex. These contributions solidify the consensus that plant fitness is intrinsically linked to its integrated microbial partnerships (Arnold et al., 2025), which we may term as the plant's “second genome” or “gut microbiome.” This Research Topic provides rigorous insights into the complex cross-talks defining the rhizobiocomplex, strengthening the foundation for studying microbially-mediated plant traits.

### Metabolic specialization and fungal hubs

Liu et al. demonstrate significant cross-talk in *Polygonatum cyrtonema*. While two forest-grown cultivars exhibited nearly identical core bacterial communities, their fungal communities differed markedly. Metabolomic analysis revealed tissue-specific signatures in tubers and roots (e.g., oleoyl ethylamide, DL-malic acid, DL-arginine), with fungal taxa showing opposing correlation patterns between the cultivars. These results underscore the vital role of the mycobiome in metabolic specialization, positioning fungi as crucial biochemical communication hubs in medicinal plants.

### Rhizobiome engineering and plant growth

Complementary research on rhizobiome engineering emphasizes that plant probiotic bacteria (PPB) inoculants do not just introduce new functional genes but also indirectly influence native rhizosphere communities by altering plant exudation and signaling pathways (Misu et al., 2025). The original research presented here demonstrates how multifunctional PPB consortia enhance nutrient solubilization, stress tolerance, and metabolic regulation, thereby fitting these activities within the larger rhizobiocomplex framework. Bright et al. isolated *Agrobacterium pusense* and *Bacillus paralicheniformis* from banana rhizospheres and demonstrated that they solubilize potassium from mica by secreting organic acids. When combined with the nitrogen-fixer *Azospirillum brasilense* and the phosphorus-solubilizer *Bacillus megaterium*, field trials achieved a 25% reduction in NPK fertilizer use while increasing bunch weight and yield. These strains also produced siderophores and exopolysaccharides, further mobilizing nutrients like zinc.

Suriani et al. showed that a consortium of *Bacillus nitrificans* and *B. velezensis* significantly boosted Robusta coffee growth, enhanced soil N–P–K levels, and raised beneficial phytochemicals (phenols, tannins, caffeine). Both strains produced indole-3-acetic acid (IAA), fixed nitrogen, solubilized phosphate, and effectively colonized roots, demonstrating strong microbe–plant communication. The application of these bioinoculants surely modulated the native microbiome in the rhizobiocomplex, ultimately affecting plant growth (Islam et al., 2023). In their article, Upadhayay et al. focused on zinc-solubilizing bacteria (ZSB), emphasizing their ability to mobilize zinc through organic acids and chelating agents, thereby increasing zinc content in grains and fruits. ZSB also exhibited other plant growth-promoting traits (P/K solubilization, ACC deaminase activity, phytohormone production), positioning them as a sustainable and affordable option for biofortification.

Salinity is a significant abiotic stress limiting plant growth. Sridhar et al. investigated a halophilic *Bacillus flexus* strain that promoted sesame growth in high-salt soils (100–200 mM NaCl). Inoculated plants showed increased levels of antioxidant enzymes (SOD, peroxidase, catalase) and decreased lipid peroxidation. These results highlight the PGPR's role in boosting induced systemic tolerance by managing osmotic balance and enhancing ROS detoxification—crucial aspects of rhizobiocomplex activity under stress.

The application of plant probiotic bacteria, whether single strains or consortia, fundamentally modulates the structure and function of the native microbiome (Islam et al., 2023), effectively engineering the rhizobiocomplex to promote plant growth. Understanding these modulations is critical for engineering the microbiome for sustainable agriculture (Misu et al., 2025).

## Conclusion

The articles in this Research Topic confirm that the rhizobiocomplex acts as a complex “external organ” for the plant. Through the precise release of exudates, plants recruit microbial partners that facilitate essential functions like nutrient solubilization and stress mitigation. Mechanisms like the rhizophagy framework suggest that plants can also actively internalize endophytic microbes or the plant gut microbiome and extract nutrients via oxidative processes. While these studies didn't explicitly test rhizophagy, observed microbial traits (e.g., mineral solubilization, ROS tolerance) strongly support broader models of plant-microbe nutrient exchange. Moving toward sustainable agriculture, we must expand focus beyond N-P-K inputs to the intricate chemical and biological cross-talks in the rhizobiocomplex. Future efforts should prioritize selecting microbial strains with superior solubilization capabilities, stress resilience, and signaling abilities for precise engineering of rhizobiocomplex. Elucidating these feedback mechanisms is crucial for developing advanced biofertilizers and microbiome-engineering strategies that enhance crop resilience and promote ecological sustainability.

## References

[B1] Arnold W. Gewirtzman J. Raymond P. A. Duguid M. C. Brodersen C. R. Brown C. . (2025). A diverse and distinct microbiome inside living trees. Nature 644, 1039–1048. doi: 10.1038/s41586-025-09316-0 40770104

[B2] Islam M. T. Hashidoko Y. Deora A. Ito T. Tahara S. (2005). suppression of damping-off disease in host plants by the rhizoplane bacterium *Lysobacter* sp. strain SB-K88 is linked to plant colonization and antibiosis against soilborne peronosporomycetes. Appl. Environ. Microbiol. 71, 3786–3796. doi: 10.1128/AEM.71.7.3786-3796.2005 16000790 PMC1169021

[B3] Islam T. Hoque M. N. Gupta D. R. Mahmud N. U. Sakif T. I. Sharpe A. G. (2023). Improvement of growth, yield and associated bacteriome of rice by the application of probiotic *Paraburkholderia* and *Delftia*. Front.Microbiol. 14:1212505. doi: 10.3389/fmicb.2023.1212505 37520368 PMC10375411

[B4] Mata D. A. D. Silva T. B. D. Macena R. A. Oliveira V. D. S. Porcino M. M. Marakaja P. B. (2025) The role of rhizophagy in nutrient, u.ptake, agricultural sustainability. Diversitas J. 10:2. 10.48017/dj.v10i2.3242.

[B5] Misu I. J. Kayess M. O. Siddiqui M. N. Gupta D. R. Islam M. N. Islam T. (2025). Microbiome engineering for sustainable rice production: strategies for biofertilization, stress tolerance, and climate resilience. Microorganisms 13:233. doi: 10.3390/microorganisms13020233 40005600 PMC11857137

[B6] Paungfoo-Lonhienne C. Rentsch D. Robatzek S. Webb R. I. Sagulenko E. Näsholm T. . (2010).Turning the table: plants consume microbes as a source of nutrients. PLoS ONE. 30:e11915. doi: 10.1371/journal.pone.0011915 PMC2912860 20689833

[B7] Paungfoo-Lonhienne C. Schmidt S. Webb R. Lonhienne T. (2013). “Rhizophagy—a new dimension of plant–microbe interactions,” in Mol. Microbial. Ecol. Rhiz, ed. F. J. de Bruijn (Hoboken, NJ: Wiley-Blackwell), 1199–1207. doi: 10.1002/9781118297674.ch115

[B8] White J. F. Kingsley K. L. Verma S. K. Kowalski K. P. (2018). Rhizophagy cycle: an oxidative process in plants for nutrient extraction from symbiotic microbes. Microorganisms 6:95. doi: 10.3390/microorganisms6030095 30227634 PMC6164190

[B9] Zhang Q. Kingsley K. L White J. F. (2022). Endophytic *Pseudomonas* sp. from *Agave palmeri* participate in the rhizophagy cycle and act as biostimulants in crop plants. Biology 11:1790. doi: 10.3390/biology11121790 36552299 PMC9775861

